# Feasibility and acceptability of point of care HIV testing in community outreach and GUM drop-in services in the North West of England: A programmatic evaluation

**DOI:** 10.1186/1471-2458-11-419

**Published:** 2011-06-01

**Authors:** Peter MacPherson, Anu Chawla, Kathy Jones, Emer Coffey, Vida Spaine, Ian Harrison, Pauline Jelliman, Penelope Phillips-Howard, Caryl Beynon, Miriam Taegtmeyer

**Affiliations:** 1 Clinical Group, Liverpool School of Tropical Medicine, Pembroke Place, L3 5QA, Liverpool, UK; 2 Wellcome Trust Tropical Centre, University of Liverpool, Pembroke Place, L69 3GF, Liverpool, UK; 3 The Liverpool Specialist Virology Centre, Royal Liverpool and Broadgreen University Hospitals NHS Trust, Prescot Street, L7 8XP, Liverpool, UK; 4 Liverpool Centre for Sexual Health, Royal Liverpool and Broadgreen University Hospitals NHS Trust, Prescot Street, L7 8XP, Liverpool, UK; 5 Liverpool NHS Primary Care Trust, 1 Arthouse Square, L1 4AZ, Liverpool, UK; 6 Liverpool Community Health NHS Trust, Wilkinson Place, L13 1FB, Liverpool, UK; 7 Centre for Public Health, Liverpool John Moores University, 15-21 Webster Street, L3 2ET, Liverpool, UK

## Abstract

**Background:**

In Liverpool, injecting drug users (IDUs), men-who-have-sex-with-men (MSM) and UK Africans experience a disproportionate burden of HIV, yet services do not reach out to these groups and late presentations continue. We set out to: increase testing uptake in targeted marginalized groups through a community and genitourinary medicine (GUM)-based point of care testing (POCT) programme; and conduct a process evaluation to examine service provider inputs and document service user perceptions of the programme.

**Methods:**

Mixed quantitative, qualitative and process evaluation methods were used. Service providers were trained to use fourth generation rapid antibody/antigen HIV tests. Existing outreach services incorporated POCT into routine practice. Clients completed a semi-structured questionnaire and focus group discussions (FGDs) were held with service providers.

**Results:**

Between September 2009 and June 2010, 953 individuals underwent POCT (GUM: 556 [59%]; community-based sites: 397 [42%]). Participants in the community were more likely to be male (p = 0.028), older (p < 0.001), of UK African origin (p < 0.001) and IDUs (p < 0.001) than participants from the GUM clinic. Seventeen new HIV diagnoses were confirmed (prevalence = 1.8%), 16 of whom were in risk exposure categories (prevalence: 16/517, 3.1%). Questionnaires and FGDs showed that clients and service providers were supportive of POCT, highlighting benefits of reaching out to marginalised communities and incorporating HIV prevention messages.

**Conclusions:**

Community and GUM clinic-based POCT for HIV was feasible and acceptable to clients and service providers in a low prevalence setting. It successfully reached target groups, many of whom would not have otherwise tested. We recommend POCT be considered among strategies to increase the uptake of HIV testing among groups who are currently underserved.

## Background

The British HIV Association [1] and other guidelines [2,3] recommend that access to HIV testing be expanded and routinely offered in non-specialist settings in order to reach out to high-risk and marginalised sections of the population and reduce late presentations. Point of care testing (POCT) is recommended in settings where venepuncture may not be possible and where there is support from a laboratory confirmatory testing service [1].

Current HIV screening relies on self-presentation to care services, resulting in missed opportunities for early diagnosis, and for preventative counselling of high-risk but currently HIV-negative individuals. United Kingdom Health Protection Agency statistics note that one quarter of the 83,000 HIV-positive individuals in the UK are unaware of their status [4] with at-risk groups particularly vulnerable to late diagnosis[5-7]. In Liverpool, three groups share a disproportionate burden of HIV infection: intravenous drug users (IDUs); men who have sex with men (MSM); and people of African origin[4,7].

This study presents findings from a study conducted in Liverpool, United Kingdom, to explore the feasibility and acceptability of POCT for HIV among at-risk and marginalised groups; the success of this approach in reaching out to target groups and previously untested individuals; and to document key factors which impact upon the implementation of POCT for HIV in order to inform others wishing to expand POCT services.

## Methods

Mixed methods of research were used incorporating participatory approaches, situational analysis, focus group discussions, analysis of routine patient monitoring data, and analysis of self-completed questionnaires patients recruited for POCT.

### Planning and Implementation of POCT for HIV

A multidisciplinary, multiagency steering group was established, comprised of clinicians, specialist HIV community nurses, members of the local primary care trust and public health researchers. Minutes, notes and correspondence following meetings were included in the analysis. Since co-authors were also participants in these groups methods that were cognisant of the observer participant approach were used. One author (PM) was an outsider to the process acting more as observer than participant. Others were active participants whose observations were also considered in the analysis. The steering group undertook situational analysis of a number of existing health and social programmes in Liverpool to identify services and programmes likely to interact with individuals at risk of HIV infection (e.g. outreach care programmes for IVDUs and sex workers) and marginalised people who may find it difficult to access health care (e.g. asylum seekers and homeless people). Services meeting these criteria were invited to participate in the POCT programme, representatives of the management were asked to join the steering group and service providers were trained in POCT for HIV. As the services identified offered a variety of health and social programmes, service providers were encouraged to develop strategies to incorporate POCT into their existing services.

To mobilise interest in POCT, an awareness-raising campaign, called Liverpool Gets Tested, was held around the week of World AIDS Day 2009 (1^st ^December). This included a number of community-based events such as HIV vigils and radio advertising campaigns.

### Service Providers' Experiences of POCT for HIV

Qualitative methods were chosen to elucidate perceptions and experiences as they allow for a more flexible and in-depth exploration of acceptability of new services. The 25 service providers performing HIV POCT at the 6 sites were invited to participate in three focus group discussions (two with providers from community sites and one with providers from the GUM site). All interviews were recorded and transcribed by the first author. A framework approach to analysis was undertaken. Qualitative analysis software (NVIVO8, QSR Software, Melbourne) was used for coding purposes. Emerging themes were grouped and triangulated with the steering committee members. Transcripts were independently reviewed by a second researcher to confirm themes.

### POCT Uptake by Service Users and a Survey of Their Perceptions of the Programme

Attendances for POCT at study sites, non-identifiable participant demographic details, and POCT for HIV results (cross-referenced with laboratory confirmatory testing where necessary) were captured by staff in logbooks. Participant demographics and self-reported clinical history were used to code a person as being in a risk exposure category for HIV or otherwise; risk exposure categories included: MSM, IDU, UK African, having bought or sold sex, having been raped, or having an HIV-positive partner. Participants could belong to multiple risk categories. Persons receiving a POCT test for HIV during the week of World AIDS Day were invited to self-complete a questionnaire about their experience of POCT.

### HIV-testing Procedures

POCT for HIV was undertaken using Determine^® ^HIV-1/2 Ag/Ab Combo (Inverness Medical Inc, UK) and tested on fingerprick blood samples. HIV testing and pre- and post-test counselling was conducted in private rooms within each site by nurses who had received training in test kit use and HIV counselling. Rooms had been previously inspected for suitability based on the presence of adequate lighting; a clean, flat and washable testing surface and accessible hand washing facilities. Participants with a reactive or invalid test (that did not resolve on immediate retesting) had a further sample sent for confirmatory same day fourth generation laboratory ELISA testing. The study nurse or clinician conveyed confirmed positive results to the participant in a follow-up appointment and referral for comprehensive HIV care was made to the HIV clinic at the Royal Liverpool Hospital, with the study clinician acting as a link person for accessing care.

### Statistical Methods

Client characteristics (logbook) and perceptions of POCT for HIV (Liverpool Gets Tested questionnaire) were expressed as proportions and compared between the LCSH and community-based sites using chi-square tests. The Wilcoxon rank sum test was used to compare differences in age distribution between the groups. Hierarchical risk exposure categories (including mutually exclusive categories with a single, or multiple risk exposures) were constructed for participants testing at the two sites and compared using Fisher's exact test. Statistical analysis used STATA v11.2 (Statacorp, College Station, Tx).

### Ethical Approval

Ethical approval was obtained from the Liverpool School of Tropical Medicine, The National Health Service (Liverpool Primary Care Trust and the Royal Liverpool University and Broadgreen Hospitals Trust).

## Results

### Planning and Implementation of POCT for HIV

Through the steering group, six sites were identified as offering health and social care services to target groups. These included a hospital-based GUM clinic (the Liverpool Centre for Sexual Health [LCSH]) and five community-based programmes (a drug-users support group; an asylum-seekers health programme; a MSM health and support programme; a travel clinic; and a support programme for homeless people).

Situational analysis of the study sites showed differences between the hospital-based site (LCSH) and the community-based sites that would influence implementation of the POCT protocol (Table 1). LCSH, an outpatient facility of the Royal Liverpool University Hospital, provides sexual health and HIV services to Liverpool and Merseyside, with over 34,000 attendances in 2009. Clients actively self-present for care and LSCH conducts limited community outreach. POCT was conducted in the department as opportunistic screening for individuals presenting for clinical care.

**Table 1:** POCT Site Characteristics

	Liverpool Centre for Sexual Health	Community-based sites
**Locations**	Hospital GUM clinic	Community sites

**Model adopted for POCT**	Opportunistic screening	Outreach case finding
	Dependent on client self-presentation to service	Dependent on service providers promoting uptake of POCT

**Groups targeted**	Patients for STI services	Drug users
	Sexual assault patients	Asylum-seekers
		MSM
		Homeless people
		Sex-workers
		Travellers
		UK Africans

**Key successes highlighted by service providers**	Rapid availability of results for traumatised clients	Outreach to encourage uptake amongst marginalised and at risk groups
	Universal screening	Giving positive and negative POCT results to clients
	Integration within nurse and healthcare assistant departmental working patterns	Opportunities for incorporating prevention messages

**Key challenges highlighted by service providers**	Time demands of POCT	Onward client referral systems
	Emotional impact of performing large number of HIV tests	Laboratory quality assurance systems
	Need to take further venous blood samples (e.g. for syphilis screening)	

The five community-based sites, although serving different client groups, had a number of common features relevant in planning the introduction of POCT. In particular, they all had existing social support programmes for the client groups they served. Therefore here it was decided to adopt a more community-orientated approach to POCT. This would involve a focus on awareness-raising about HIV and community mobilisation (including organising church vigils and radio campaigns) to encourage uptake of POCT in target and marginalised groups.

### POCT Uptake by Service Users and Their Perceptions of the Programme

Between September 2009 and June 2010, 953 participants underwent POCT for HIV, 556 (58%) at LCSH and 397 (42%) at the community-based sites. During the Liverpool Gets Tested Campaign, 154 people underwent POCT for HIV. Of individuals tested, and where data were recorded, 659/946 (70%) were male and the median age was 29 years (interquartile range (IQR): 23 - 38). A greater proportion of people testing in the community-based sites (74%) were males compared to in LCSH (67%, p = 0.028). However, the proportion of males who self-identified as MSM was higher at LCSH (36%) compared to the community-based sites (44%, p = 0.050)

Complete risk category data was available for 927 (97%) participants, with all missing data from those testing at community-based sites (Table 2). In total, 475 (51%) were found to be in one HIV risk category. A further 41 (4%) participants were in two risk categories and 2 (0.2%) were in three risk categories. The most frequently reported category was MSM (28%), followed by UK African origin (14%) and current or previous intravenous drug use (8%). Reflecting the clients groups served by the two models of POCT (community and hospital-based), participants tested in the community-based sites were more likely to be UK Africans and to report current or previous IDU compared to those tested in LCSH. In contrast, participants tested at the hospital-based LCSH were more likely to have reported having been raped and having been exposed to an HIV-positive partner.

**Table 2:** Risk categories of participants undergoing POCT and completing "Liverpool Gets Tested Questionnaire" at community-based sites and LCSH

	Total (%)	Community-based site (%)	LCSH (%)	P-value†
	*n = 927*	*n = 371*	*n = 556*	

Exposure Categories*				
Mutually exclusive: one case is represented in ONLY one category)^§^				
MSM				
*MSM only*	245 (26.4)	95 (25.6)	150 (27.0)	0.649
*MSM & UK African*	3 (0.3)	2 (0.5)	1 (0.2)	0.568
*MSM & bought or sold sex*	3 (0.3)	1 (0.3)	2 (0.4	0.648
*MSM & HIV positive partner*	7 (0.8)	1 (0.3)	6 (1.1)	0.253
*MSM & reported rape*	4 (0.4)	0 (0.0)	4 (0.7)	0.154
*MSM & HIV positive partner & reported rape*	1 (0.1)	1 (0.3)	0 (0.0)	0.400
*MSM & bought or sold sex & HIV positive partner*	1 (0.1)	1 (0.3)	0 (0.0)	0.400
UK African				
*UK African only*	116 (12.5)	77 (20.8)	39 (7.0)	< 0.001
*UK African & bought or sold sex*	3 (0.3)	0 (0.0)	3 (0.5)	0.279
*UK African and HIV-positive partner*	5 (0.5)	0 (0.0)	5 (0.9)	0.164
*UK African & reported rape*	6 (0.6)	2 (0.5)	4 (0.7)	1.000
IVDU				
*IVDU only*	72 (7.8)	67 (18.1)	5 (0.9)	< 0.001
*IVDU & bought or sold sex*	10 (1.1)	10 (2.7)	0 (0.0)	< 0.001
Bought or sold sex				
*Bought or sold sex only*	21 (2.3)	4 (1.1)	17 (3.1)	0.069
HIV-positive partner				
*HIV-positive partner only*	14 (1.5)	1 (0.3)	13 (2.3)	0.011
Reported rape				
*Reported rape only*	7 (0.8)	0 (0.0)	7 (1.3)	0.046

*Summary Categories^±^:*				
NOT mutually exclusive: one case can be represented in multiple categories				
Any MSM	264 (28.5)	99 (26.7)	165 (29.7)	0.277
Any UK African	133 (14.3)	81 (21.8)	52 (9.4)	< 0.001
Any IVDU	82 (8.8)	77 (20.8)	5 (0.9)	< 0.001
Any bought or sold sex	38 (4.1)	15 (4.0)	23 (4.1)	0.944
Any HIV-positive partner	28 (3.0)	2 (0.5)	26 (4.7)	< 0.001
Any reported rape	18 (1.9)	2 (0.5)	16 (2.9)	0.011

* Where data on exposure category captured

^§ ^Risk categories are grouped based on hierarchical categories. Any one person with multiple risks may ONLY be represented in the highest category

^± ^These groups presented are NOT mutually exclusive, meaning a case can be represented in multiple groupings. These summarised categories are meant to give a broader picture of the exposure categories and will NOT add up to the overall total number of participants

^† ^Fisher's exact test

Seventeen confirmed HIV diagnoses were made (prevalence = 1.8%); 14 from LCSH (prevalence = 2.5%) and three from community based sites (prevalence = 0.8%). Ten individuals diagnosed HIV-positive were MSM, four were UK Africans, one reported IDU and one reported having been gang raped. One HIV-positive participant had no identified risk category by our definition. HIV prevalence in those in a risk category was 16/517 (3.1%). All but one are under on-going follow up in clinical settings. The remaining one is an IDU with a chaotic lifestyle who has repeatedly failed to attend appointments but remains in the Liverpool area.

During the Liverpool Gets Tested Campaign, 128/154 (83%) of individuals participating in POCT for HIV completed a questionnaire. Risk exposure among those completing questionnaires categories were similar to that of the overall study population, expecting that no participants who reported having been raped or having an HIV-positive partner completed a questionnaire. Approximately half (65/127, 51%) had never tested for HIV before and when questioned about their experience of POCT 105/125 (84%) preferred POCT to laboratory testing and 115/125 (92%) stated they would recommend POCT to others. There were no differences in reported previous testing for HIV (p = 0.867) or preference for POCT between LCSH and the community-based sites (p = 0.759). However, a greater proportion of participants testing at community-based sites (96%) stated that they would recommend POCT compared to those testing at LCSH (82%, p = 0.015).

### Process Evaluation of Implementation and Training

Evaluation of minutes of steering group meetings during preparation for and implementation of POCT revealed a number of issues important to others considering multi-disciplinary POCT for HIV programmes. The principal concern raised by service providers was that POCT would impact upon their regular duties. This overlapped with concerns from healthcare commissioners that POCT for HIV could distract from core service provision. Furthermore, questions were raised as to how POCT for HIV would continue to be funded following the completion of the research project.

The competing demands of the health care commissioners to demonstrate value for money of POCT and that of research academics to demonstrate effectiveness of the HIV testing programme required considerable time and mutual co-operation to resolve. Demonstration that new HIV-positive diagnoses were being made amongst marginalised groups such asylum-seekers and drug users helped persuade commissioners of the value of the POCT.

### Service Providers' Experiences of POCT for HIV

Through analysis of focus group discussions, we identified key themes raised by service-providers in LCSH and the community-based sites about their experiences of participating in the POCT for HIV programme.

#### Community-based service providers

Health care providers working within community-based sites highlighted the benefits of reaching out into the community to increase awareness of POCT for HIV and engaging individuals who may have otherwise not been able to access health services:

*"When people come to [the GUM clinic], they already know a certain amount about [HIV] and have thought about it but when we reach out we are tapping out into a whole different community"*. Nurse, male, drug users outreach programme

The opportunities to integrate POCT with prevention messages for difficult-to-reach clients was noted by service providers who felt that POCT provided a unique opportunity for integrating public health messages:

*"They were high risk patients that were non-reactive so it was a good opportunity to reinforce behavioural change ... just reinforce the fact that if he is going to have sex outside of his marriage, particular with high risk, then he needs to protect himself because his whole world will just sort of collapse. So it was worth doing it for that." *Community health worker, male, MSM outreach project

A number of challenges associated with integrating POCT into community-based service delivery were identified. In particular service providers felt there was a careful line to tread between supporting someone in a marginalised and vulnerable position to learn their HIV-status and placing undue pressure on an individual who had not planned to take an HIV test.

External quality assurance systems were identified as a key area requiring a strengthened support system. Community-based service providers felt if they obtained reactive or invalid results on POCT, it was difficult to communicate with laboratory personnel and often relied upon informal communications. Delays in obtaining a final HIV result created a period of "diagnostic limbo" for service providers and clients, which negatively impacted upon their relationship.

#### LCSH-based service providers

Service providers within LCSH highlighted the usefulness of POCT for individuals who were victims of sexual assault and who were about to receive post-exposure prophylaxis. They felt confident offering clients negative results, facilitating rapid reassurance for individuals suffering traumatic experiences. They also acknowledged the benefits of being able to give a rapid result and not having to worry about clients failing to return to the department to collect results.

LCSH integrated POCT into departmental service delivery using a different model to the community-based sites. Nurses and healthcare assistants (HCAs) undertaking consultations with outpatient clients would offer POCT. If the client agreed, the health care assistants (HCAs)/staff nurses would perform the finger-prick tests themselves. The more complex patients (such as sexual assault cases) were referred to the health advisers. This allowed nurses and HCAs to perform more tests and maximise their busy workload, however, health advisers reported feeling overwhelmed by the difficult cases. We noted that health advisers identified all reactive results in the LCSH and one commented on the impact of dealing with traumatised clients:

*"For us it's doing the same procedure again and again and again and everybody that comes has got a different journey to the test. Some of them are incredibly traumatic. Seen a man that was raped yesterday. I've got a prisoner coming in whose been raped. [...] That's the run of the mill for us. It's just too many people." *Health advisor, female, LCSH

##### Issues identified by service-providers in both LCSH and community-based sites

A common theme for both groups of service providers was their initial concern and confidence in their knowledge of HIV should clients ask difficult questions. These concerns overlapped with the experience of undertaking an activity that was seen as something new and outside their realm of usual practice. Whilst most service providers were experienced in technical procedures and quickly became confident in doing finger-prick tests, many had concerns about how to deal with a positive HIV result and expressed fear at the prospect of giving a result that could have profound and long-lasting implications for the client's health and social life. However, service providers who had given reactive results expressed that they were pleased to enable clients to take control of their illness.

"I think its really good for the nurse or whoever is doing the test to give somebody the result instantly. Because you feel like you are actually [...] helping them achieve something positive, whether it is a negative or a reactive result."

Health worker, male, asylum-seekers health programme

Similarly, the giving of negative results had a profound effect on service providers. As confidence and experience grew, service providers recognised the importance of the relationship between the client and themselves and how a negative POCT result represented an opportunity to reinforce educational messages and empower clients to change high-risk behaviour.

Another key theme identified was the speed with which results were obtained. Service providers noted that clients may not have attended hospital or the community site expecting to undertake a HIV test and waiting 20 minutes for the results provided little extra time to receive counseling and understand the implications of the result. Service providers thus found themselves reigning back their enthusiasm for offering clients POCT, in order to ensure clients understood and had time to consider the possible implications:

*"Doing the last few tests that I have been doing, I've been saying: look, you are going to get a result quite quickly today you know. Are you prepared for that?" *Nurse, female, travel clinic

## Discussion

This community- and GUM clinic-based programme has demonstrated that POCT for HIV can reach high-risk, previously marginalised groups, including UK Africans, MSM, homeless people, sex-workers and IDUs, with 953 tests being conducted within a 10 month period, over half of which were among high-risk groups. Of those who had a POCT for HIV during World AIDS Week, over fifty percent had never previously undergone HIV testing.

POCT for HIV has been successfully scaled-up in high prevalence HIV settings (particularly in sub-Saharan Africa), through voluntary counselling and testing (VCT)[8] programmes that provide opportunities to incorporate HIV prevention messages into the testing process[9]. Our findings suggest that POCT in the UK could facilitate discussion of HIV prevention messages, particularly for individuals who are in high-risk categories and who test negative for HIV.

With a third of new UK HIV diagnoses being identified with a CD4 count of less than 200 cells/ul [4], this project demonstrates the potential of incorporating POCT into existing hospital and community based services to increase the uptake of HIV testing, decrease late presentations and increase access for marginalised groups. Late presentation is associated with higher mortality[10,11], prolonged hospital stays and increased hospital-related costs[12].

This study used 4^th ^generation rapid antibody/antigen tests for HIV. Previous studies in the UK and elsewhere that have introduced POCT to hospital[13-18], primary care[13,19] and community settings[20-25] have used antibody-only tests. Although 4^th ^generation tests have the advantage of a shortened window-period and increased sensitivity and specificity[26], we did not identify any seroconverters on the basis of p24 antigen and did have two false positive p24 antigen results. Our findings, which are being presented elsewhere, suggest that 4^th ^generation test kits do not have an advantage over existing 3^rd ^generation tests for a community-based screening programme.

We found that service providers and clients were accepting of POCT. The pre-selection of study sites to identify services that would be most successful in providing POCT may have influenced the high observed acceptability for clients and service providers. Previous studies investigating community-based POCT for HIV in the UK raised concerns that lack of confidentiality and stigma surrounding HIV may hinder service uptake[27]. However, we found participants (over 90%) would recommend POCT to others with a greater proportion of participants from community sites than in the GUM setting making the recommendation. There are a number of possible explanations for this. Firstly, the differing client groups (and with potential different spectrum of reasons for presentation) tested may have meant that there was differing levels of what to expect from POCT prior to testing. Alternatively, the way in which testing was offered may have influenced experience of POCT. In the community, service providers, who may have known the client previously and have a long-standing trusting and supportive relationship with them, offered POCT. In contrast, in the hospital, POCT was likely to be offered by a service provider whom the client was meeting for the first time in the context of a busy GUM clinic appointment.

We also describe positive experiences among service providers, particularly regarding an increase in knowledge and awareness of needs within the community. We attribute this to the decentralised approach taken to allow service providers to integrate POCT into their pre-existing community-programmes.

Establishment of the POCT steering committee and involvement of health-care commissioners in management decisions was critical to the successful scale up. Although we did not assess in this study, it is possible that the availability of POCT may have discouraged some potential testers, Furthers studies to examine the perceptions of individuals who refuse HIV testing towards POCT are required.

In the hospital GUM clinic setting, a large numbers of screening tests were undertaken in a time-pressured environment. As a consequence clients perceived to be "more difficult" were tested by health advisers instead of the HCAs or staff nurses. The emotional impact for service providers of undertaking HIV testing is well recognised[19,23]. We found that the visual emergence of the HIV result to both the client and the service provider to be a difficult experience for service providers. It is important to ensure support and debrief mechanisms exist for staff undertaking rapid HIV testing.

Certain caveats are noted within our study; our participants may represent the most vocal population with particularly strong feelings, either positive or negative, about the service. The high uptake of questionnaire completion (82.5%) during the World AIDS Day study period and our measures to ensure that clients completed questionnaires in private and submitted anonymously attempted to mitigate this. Furthermore, a recognised limitation of focus group discussions is that group dynamics may lead to over-emphasis of certain themes which, in individual interviews, may not have been particularly important. We limited this effect by structuring groups to include service providers working from teams in similar environments and by using flipchart exercises to focus group discussions.

## Conclusions

We found POCT to be acceptable to service providers and clients. The decentralised rolling-out of POCT was successfully integrated into a broad range of existing services, and reached diverse groups of clients, including targeted groups. Further cost-effectiveness studies and epidemiological studies are required to assess the additional benefit of 4^th ^generation POCT over current testing strategies. Together these findings will have implications for further scaling-up of POCT services in Liverpool and more widely in the UK, and for the introduction of additional, novel, point of care tests.

## Competing interests

The authors declare that they have no competing interests.

## Authors' contributions

PM, AC, KJ, EC and MJ conceived of the study and participated in its design. AC supervised laboratory virological services. VS, IH and PJ undertook data collection. PM and MT undertook data management and statistical analysis. PM, MT, PPH and. CB undertook qualitative data analysis and programmatic evaluation. PM, MT, PPH and CB drafted the paper. All authors read and approved the final manuscript.

## Funding

PM is supported by the Wellcome Trust Clinical PhD Programme at Liverpool School of Tropical Medicine

Gilead UK and Ireland fellowship programme paid for salary for VS and funded test kits

## Pre-publication history

The pre-publication history for this paper can be accessed here:

http://www.biomedcentral.com/1471-2458/11/419/prepub
